# Flexible Graphite-on-Paper Piezoresistive Sensors

**DOI:** 10.3390/s120506685

**Published:** 2012-05-22

**Authors:** Tian-Ling Ren, He Tian, Dan Xie, Yi Yang

**Affiliations:** 1 Institute of Microelectronics, Tsinghua University, Beijing 100084, China; E-Mails: tianh10@mails.tsinghua.edu.cn (H.T.); xiedan@tsinghua.edu.cn (D.X.); yiyang@tsinghua.edu.cn (Y.Y.); 2 Tsinghua National Laboratory for Information Science and Technology (TNList), Tsinghua University, Beijing 100084, China

**Keywords:** flexible, paper-based, graphite, piezoresistive devices

## Abstract

We demonstrate novel graphite-on-paper piezoresistive devices. The graphite was used as sensing component. The fabrication process can be finished in a short time with simple tools (e.g., a scissor and a pencil). A small array of six paper-based piezoresistive devices is made. The whole device is flexible. The test results showed that the change of resistance was proportional to the applied force. A paper-based weighing balance was also made as an example of applications. This novel array of paper-based piezoresistive devices will open wide applications in force and acceleration sensing areas.

## 1. Introduction

Flexible sensors and actuators are the cutting edge technology [1–17], which can interface well with humans and have wide applications in the medicine and biology areas. Conventional CMOS technology [18] uses silicon as the substrate, but the original silicon substrate can't be flexible. In order to be flexible, chemical-mechanical polishing is used to reduce the silicon substrate to submicron thickness [19], however this method is challenging and high cost. Despite recent attempts at utilizing polymer materials as flexible substrates [20–24], the cost of these materials is still relatively high. In addition, the fabrication process needs to be done in clean room and takes several days. The high cost and complex process are the main drawbacks which have limited the applications and markets for flexible devices, so it is desirable to find a new low cost substrate material and develop a new process to easily fabricate flexible devices.

Due to the softness, lost cost and lightweight properties of paper materials, flexible devices can be made from them easily, such as paper-based sound source devices [25], microfluidics systems [26], *etc.* This may open a new venue of technologies. In particular Liu *et al.* have made pioneering contributions making paper-based sensors [27] to be inexpensive, simple to fabricate, lightweight, and disposable. Inspired by the work of the reference [27], we decide to conduct an in-depth study to improve the device performance to become more inexpensive, simpler to fabricate, more lightweight, and more disposable.

Here, we chose A4 paper (88 μm thick) as the substrate and developed a new process to fabricate flexible piezoresistive devices in a short time. Graphite is used as the sensing component. In order to realize good control of the shape of carbon resistor, pencil marks are directly drawn on the paper. In order to realize good electrical connections and shaped electrodes, copper foil was used as the electrode material. The fabrication process was reduced into less than half an hour of work with simple tools. The cost is just $0.01 per device. A small array of six paper-based force sensors is made. The whole device is flexible. A paper-based weighting balance is also made based on the same idea.

## 2. Experimental Section

### 2.1. Working Principle

The working principle of the paper-based force sensor is based on the piezoresistive effect. The conventional silicon-based force sensor usually uses a cantilever beam inducing strain/stress. Based on the piezoresistive effect, the strain/stress can be converted into a resistance change by the sensing component, which is easily detected by the electrical instrument. In order to realize a flexible system, a paper substrate is used instead. The paper cantilever beam is made to let the force apply on it. The graphite located on the cantilever beam functions as the sensing component. When force is applied on the cantilever beam, the stress of the beam will change sensitively.

### 2.2. Device Structure

**Figure 1.**
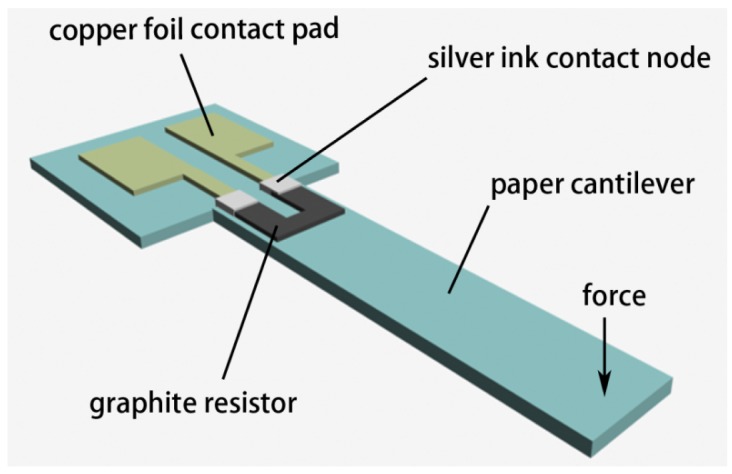
Schematic structure of a paper-based force sensor. Graphite is used as the sensing component.

The schematic structure of the paper-based force sensor is shown in Figure 1. In this structure, a graphite resistor is located at the root of the cantilever beam. When a force is applied to the beam structure, the graphite resistor will experience a mechanical strain/stress, which then induces a change in the resistance of the resistor. Measuring the change in resistance can reflect the magnitude of the applied force.

### 2.3. Fabrication Process

**Figure 2.**
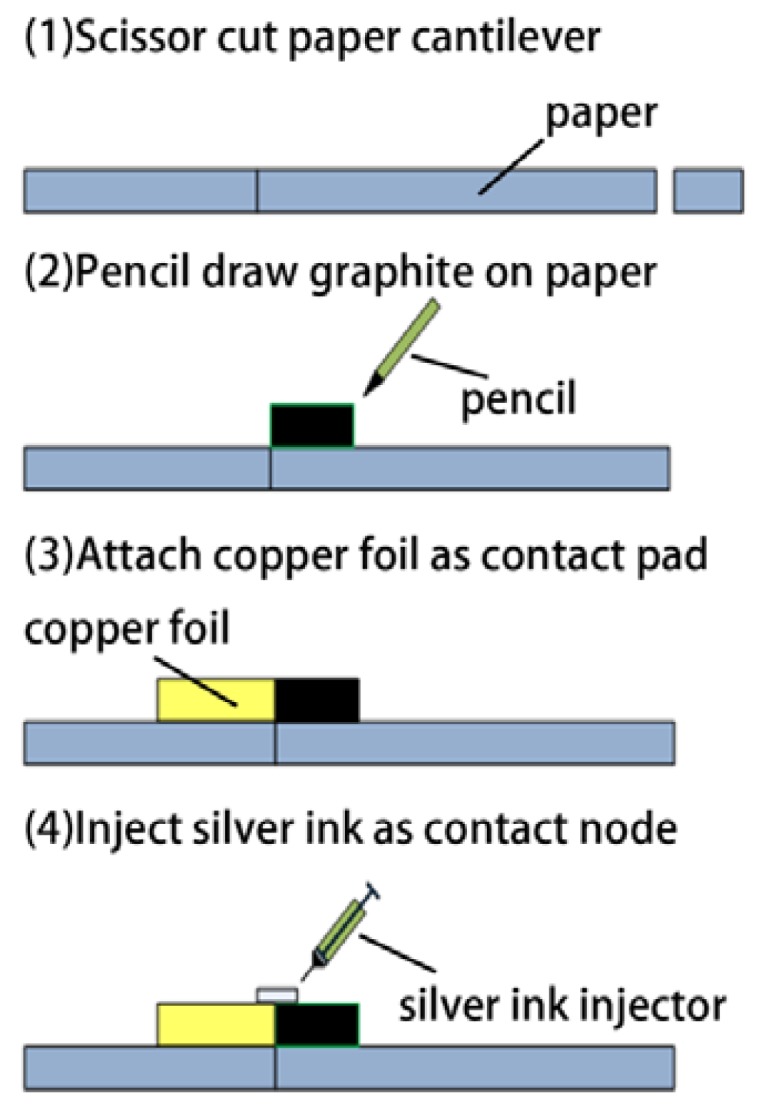
Fabrication process of the paper-based force sensors.

**Figure 3.**
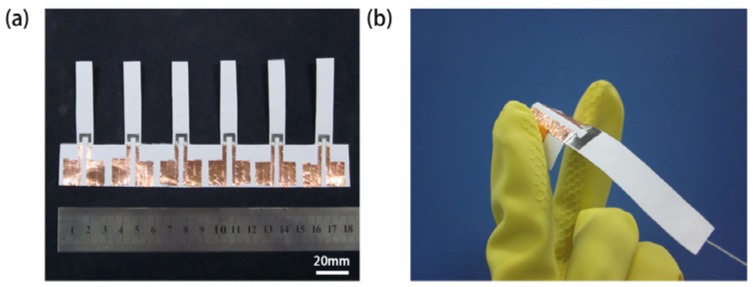
A photograph of paper-based piezoresistive devices. (**a**) A small array of six paper-based piezoresistive devices; and (**b**) A flexible paper-based piezoresistive device.

The fabrication process of the paper-based force sensors is shown in Figure 2. Firstly, A4 paper (88 μm thick) is used as the original substrate material. We fabricated paper cantilever beams by cutting the paper using scissors. We manually drew graphite resistors using B2 pencil, and applied contact pads using low-resistivity copper foil. Silver ink is used to enhance the electrical connection between the graphite resistors and the copper foil. The paper device was baked at 80 °C for 30 minutes. Fabricating a small array of six paper-based force sensors (Figure 3) typically takes less than one hour.

### 2.4. Test Platform

**Figure 4.**
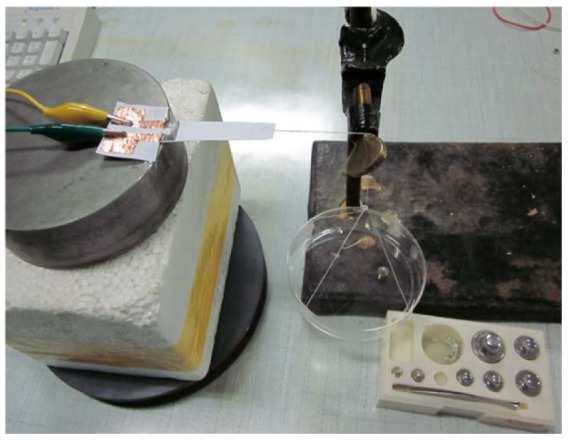
The test bench of the paper-based piezoresistive devices.

In order to apply force, we attach a wire to the cantilever beam. As shown in Figure 4, the wire connects with a plastic box, which can support weights. In order to detect the change in resistance of the sensor, a Keithley 2400 source meter is used for signal processing in our piezoresistive sensing systems.

## 3. Results and Discussion

### 3.1. Mechanical Properties of the Paper Cantilever Beam

**Figure 5.**
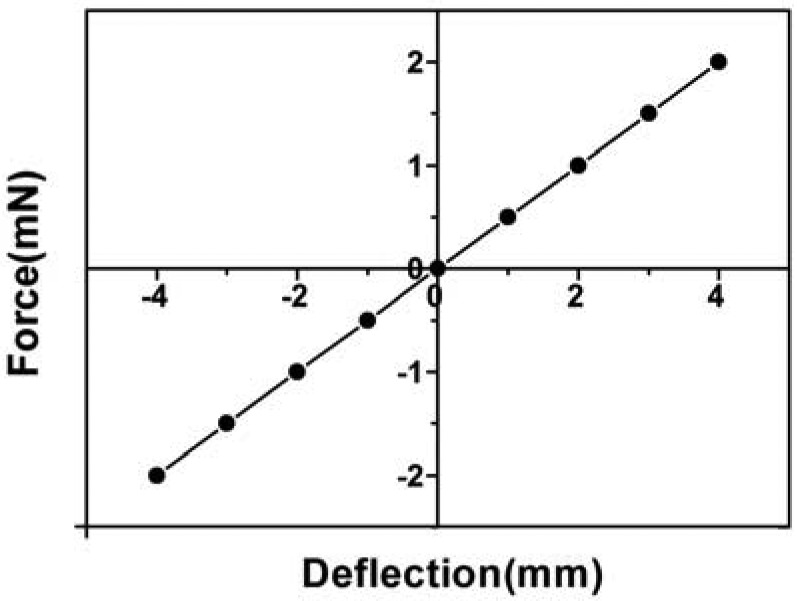
The mechanical properties of the paper cantilever beam.

The dimensions of the paper cantilever beam are 45 mm long, 8 mm wide and 88 μm thick. As shown in Figure 5, forces applied to the free end of a paper cantilever are measured as a function of the beam deflections. The stiffness of the paper cantilever was calculated to be 0.5 mN/mm. The Young's modulus of the paper material can also be calculated by using the beam equation [27]:

(1) $$\text{E}=\frac{4L^{3}F}{\delta WH^{3}}$$

where *E* is the Young's modulus of the paper material, *F* is the applied force, *δ* is the beam deflection, and *E*, *W*, and *H* are length, width, and thickness of the paper cantilever beam. The Young's modulus of the paper material was calculated to be 0.03 GPa, which is 5,000 times lower than that of silicon (typically, 150 GPa for single crystal silicon).

### 3.2. Electrical Properties of the Carbon Resistor

**Figure 6.**
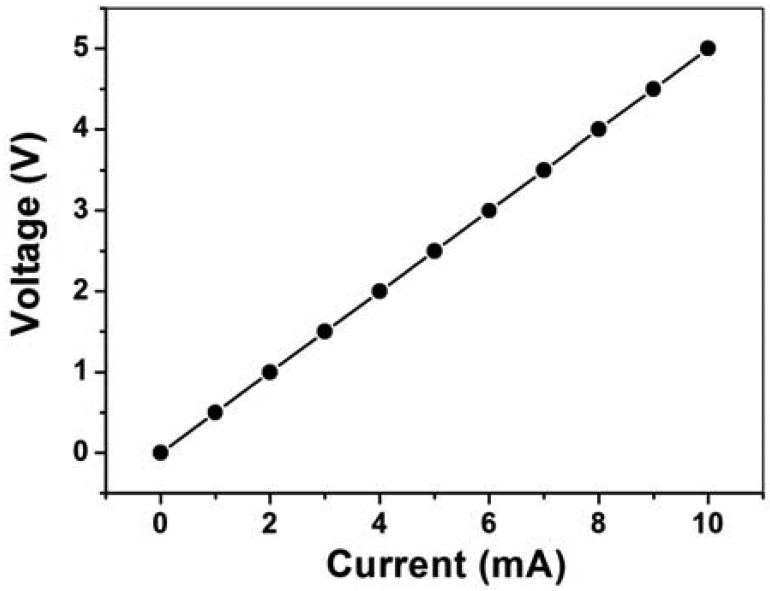
The electrical properties of the carbon resistor.

The current-voltage (I-V) characteristic of the carbon resistors was measured using the Keithley 2400 source meter under ambient conditions (25 °C temperature and 50% relative humidity). As shown in Figure 6, the measured resistors reveal a linear, ohmic I-V behavior, and the resistance of the resistors is 500 Ω.

### 3.3. Paper-Based Force Sensor

**Figure 7.**
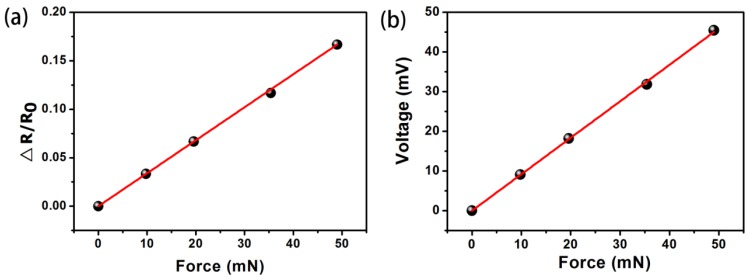
The test results of paper-based piezoresistive devices. (**a**) The plot of the relative resistance change *vs.* the applied force. (**b**) The plot of the output voltage *vs.* the applied force.

Figure 7(a) shows a sensitivity curve of resistance as a function of the applied force. The range of force measurement was 50 mN, and the resolution of force measurement was 500 μN. In order to convert the change in resistance of the sensor into a more readable electrical signal (voltage), a Wheatstone bridge circuit is commonly used for signal processing in MEMS piezoresistive sensing systems. Figure 7(b) illustrates a calibration curve of the paper-based sensor with an integrated Wheatstone bridge circuit. The operate voltage of the Wheatstone bridge circuit is 5 V. The sensitivity of the sensor was 0.9 mV/mN.

### 3.4. Paper-Based Weighting Balance

**Figure 8.**
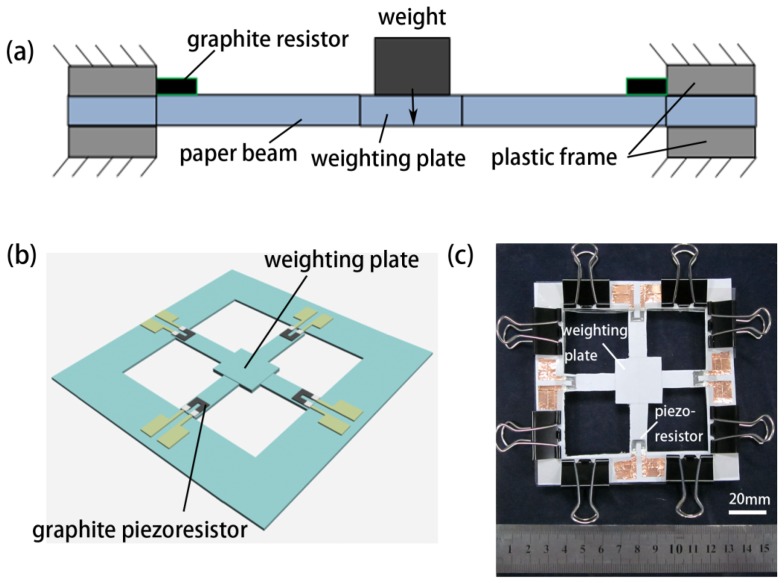
A paper-based weighting balance. (**a**) Schematic cross section of the device; (**b**) Schematic 3D structure of the device; and (**c**) Photograph of the device.

A paper-based weighting balance is also developed using the same working principle. As shown in Figure 8(a), four sides of paper-based force sensing beams can combine together to form a weighing balance. When an object was placed on the top of the weighting plate, its gravity will induce stress/stain on the sensing beams. Figure 8(b) is the top view photograph of a balance prototype, where four force sensing beams are combined together. An actual image of this prototype is shown in Figure 8(c).

**Figure 9.**
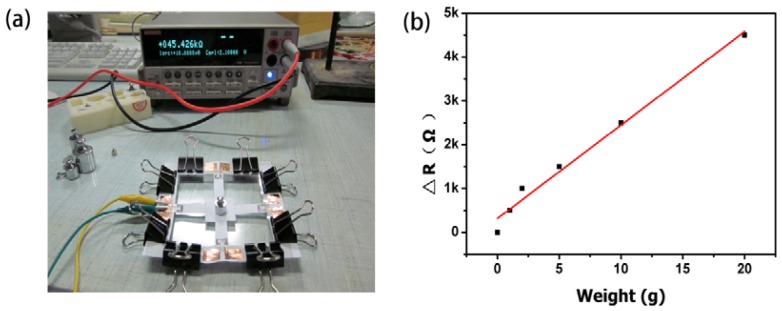
Test bench and test results of paper-based weighting balance. (**a**) The test bench is used to test the change of resistance when the device is applied with different weighting magnitude; and (**b**) Plot of the relative resistance change *vs.* the applied weight.

We calibrated the balance by measuring the change in resistance of the graphite resistor from one sensing beam as a function of the applied weight (Figure 9(a)). As shown in Figure 9(b), the measurement range of the balance was 20 g, and the measurement resolution was 50 mg.

### 3.5. Comparison of Our Paper-Based Sensor with Other's Work

Compared with the Liu's pioneering work using paper in reference [27], we have made in-depth work to improve the device to become more inexpensive, simpler to fabricate, more lightweight, and more disposable. We have listed the originality of this work compared with the previous studies in Table 1.

## 4. Conclusions

In this work, we have demonstrated flexible paper-based force sensors. A4 paper (88 μm thick) was used as the substrate. Graphite was used as the sensing component. In order to realize good control of the shape of carbon resistor, pencil traces were directly drawn on the paper. In order to realize good electrical connection and shaped electrodes, copper foil was used. The fabrication process can be finished in a short time with simple tools (e.g., scissors and a pencil), without the need of a clean room. The cost is just $0.01 per device. A small array of six paper-based piezoresistive devices was made. The whole device was flexible. The test results show that the change of resistance was proportional to the force applied. A paper-based weighting balance was also made as an example of applications. This novel array of paper-based piezoresistive devices should open wide applications in the force and acceleration sensing areas.

## Figures and Tables

**Table 1.** Comparison of specifications of our paper-based MEMS sensor to the Liu's work in Reference [27].

**Specifications**	**Paper-Based MEMS Sensor (This Work)**	**Paper-Based MEMS Sensor (Reference** [27])
Substrate	A4 paper (88 μm thick)	Whatman 3MM chromatography paper (340 mm thick)
Parrten method for substrate	Scissor	Laser equipment
Beam size (L × W × H)	45 mm × 8 mm × 0.088 mm	44.5 mm × 7.7 mm × 0.34 mm
Beam stiffness	0.5 mN·mm^−1^	2 mN·mm^−1^
Natural frequency	∼15 Hz	∼25 Hz
Force range	50 mN	16 mN
Force resolution	500 μN	120 μN
Sensitivity	0.9 mV/mN	0.84 mV/mN
Sensing material	Graphite pencil	Graphite ink
Parrten method for sensing material	Directly draw carbon resistor by using pencil	Screen print carbon resistor by using stencil
Electrode material	Copper foil	Silver ink
Fabrication process	<half an hour in any place	<1 hour in laboratory
Device cost	$0.01 per device	$0.04 per device
